# Diseases of Nerves and Electrical Treatment

**Published:** 1905-01-21

**Authors:** 


### DISEASES OF NERVES AND ELECTRICAL TREATMENT.

**Paralysis of the Palmar Branch of the Ulnar.**—T. Patmore[1] records an instance due to a blow on the hand. The patient could grasp large objects but not small ones such as pins. Besides the usual wasting of the muscles of the hand, the clawing of the inner fingers, and the inability to separate or adduct the fingers, it was noted that the thumb was adducted chiefly by the action of the extensor secundis internodii, and that sensation nowhere seemed to be lost.

**Brachial Plexus.**—Much discussion has taken place as to the formation of the different cords of this plexus and the spinal representation of the arm muscles. R. T. Williamson[2] describes two cases of paralysis affecting not only the deltoid, biceps, brachialis, and supinator longus, but also the triceps and the extensors of the wrist and fingers. Now Erb's paralysis from lesions of the fifth and sixth cervical roots affects the first four muscles, and he suggests that this wider paralysis is due to a lesion of the upper cord when composed of the cervical nerves five, six, and seven, or a lesion of all these nerves separately. Wilfrid Harris has, however, shown that variations occur in the position of the plexus, and that it may be shifted up or down as much as half a root (prefixed or postfixed). Thus the above paralysis may be due in a prefixed plexus to a lesion of roots five and six only. It may be noted that Harris, in his original paper, insisted that Erb's paralysis is due to a lesion of the fifth nerve only and in two cases he cut across the fifth root and sewed it into a lower and intact one, the sixth, and obtained a cure. L. Pierce Clarke[3] considers that this "brachial birth palsy" is due chiefly to tension on the fifth and sixth cervical roots, but it may include the seventh and even the eighth and first dorsal; there is rupture of the nerve fibres and overgrowth of connective tissue between the torn ends. This generally occurs through forcible depression of the shoulder, while the head is bent to the opposite side and rotated. It is desirable to operate and suture the sound nerve ends as early as possible. This should be followed by electrical and similar treatment. Terriberry pointed out that many cases were not a result of stretching the nerves, and referred to one produced by pressure of the blades of the forceps upon the nerve roots, which completely recovered. In another, a breech presentation, there was double Erb's paralysis, also due to pressure, and this too improved rapidly. The question of the actual nature of the lesion in each case is important, and also the time at which an operation should be undertaken. As other speakers remarked, though on purely surgical grounds it might be desirable to operate within the first few months of life, yet many cases improve and at present the risk of operations in early life is very great. Cases of brachial neuritis involving the fifth and sixth nerves have been reported by Simon, and another by C. J. Cattle.[4] The latter was in a miner, and not due to traumatism, but probably to continuous work with the arms raised above the head causing contractions of the scalenus medius through which these nerves pass. The paresis at first took the usual Erb's type, but the flexors of the elbow quickly recovered. Cattle points out that the fifth nerve first gives a branch to the rhomboids, which, however, probably consists of fibres derived from the fourth nerve. Then a branch, joining one from the sixth, supplies the serratus magnus. Afterwards it can be divided into an upper and a lower fasciculus, the former supplying the shoulder muscles and the latter the elbow muscles. The injury here seems to have chiefly affected this upper fasciculus on the left side, for the deltoid and spinati remained wasted after six months of galvanic treatment. On the right side the infra-spinatus remained in the same state, and the serratus magnus also failed to recover which pointed to an injury somewhat higher up. Other writers have noticed the frequency of injury to these nerves among miners, as a result of the cramped position in which they work.

**High-frequency Currents.**—J. A. Codd[5] has employed high-frequency currents in severe or intractable neuralgias and myalgias. He connects the bottom of the resonator to the condensation plate and directs the effleuve to the diseased part, and in some cases gives autocondensation as well. The pain in severe sciatica or lumbago usually ceases for many hours after the first sitting, and as the applications are continued the intermissions are longer and the severity decreases till it ceases altogether. In some cases of tinnitus aurium very good results followed, but success was not invariable. Improvement was noted in diabetes, pruritus ani and piles, and two cases of vomiting from anorexia nervosa were cured. T. J. Bokenham[6] claims that Doumer's high-frequency treatment is very successful in sphincter fissures, and in the small fissures associated with piles, and in relieving pruritus. It is most valuable, too, for piles in the early stages, but when acute conditions or much thickening from old-standing trouble exists the results are less certain and rapid. The writer uses a high-vacuum electrode and a small current of 100 to 150 milliampères. With a metal electrode, currents three or four times as strong may be employed. In 13 cases of simple fissure all were quickly cured, and in 25 cases of recent piles 14, while 9 were relieved from all discomfort. Fifteen instances of pruritus ani were also cured promptly. The cure of the piles could not be due to stretching, because the electrodes used were too small to act in such a way—viz., from 3 to 15 millimètres in diameter.

**Death from Electric Shock.**—T. Oliver[7] discusses the dangers of the live rail in electrical railways, and describes the case of a man who fell upon one at Wallsend. The patient was convulsed and remained unconscious for two hours. The right foot was burned and deep ulcers formed, which showed no tendency to heal a month after the accident. Grange in 1885 held that death is due to hæmorrhages in the medulla. D'Arsonval thought that destruction of the tissues and inhibition of the nerve centres took place, and in the latter case death would be due to arrest of the respiration. Tatum, on the other hand, showed reason for referring it to paralysis of the cardiac wall, but in electrocutions at New York under alternating currents of 1,500 volts the heart was found to beat and respiration to continue after short contacts. Oliver himself in 1898 found that the heart usually stopped first, though with very high voltages there might be simultaneous arrest of both the heart and breathing, but he never succeeded in producing primary arrest of the respiration. Prevost and Battelli confirmed this with regard to currents of moderate tension, and R. H. Cunningham concluded that the respiratory centre was not destroyed but that the left heart was chiefly affected. The voltage fatal to human beings depends more on the conditions of contact than on its amount. Contact with 2,000 volts has not been fatal, while in the Lambeth bath case contact by wet hands caused death though the current was only 250 volts. The danger is greatest when one part of the body is in contact with the earth and another with the live rail. There is also a great difference between the effects of continuous and alternating currents. After an accident, artificial respiration for half an hour should be carried out, and Cunningham recommends transfusion of defibrinated blood.

[1] Brit. Med. Jour., Mar. 12. [2] Lancet, Aug. 13. [3] Jour. Nervous and Ment. Dis., Oct. [4] Brit. Med. Jour., Mar. 12. [5] Ibid, July 23. [6] Lancet, July 2. [7] Lancet, Aug. 20.

